# Creating a Theoretical Framework to Underpin Discourse Assessment and Intervention in Aphasia

**DOI:** 10.3390/brainsci11020183

**Published:** 2021-02-02

**Authors:** Lucy Dipper, Jane Marshall, Mary Boyle, Deborah Hersh, Nicola Botting, Madeline Cruice

**Affiliations:** 1 Division of Language and Communication Science, School of Health Sciences, University of London, London EC1V 0HB, UK; J.Marshall@city.ac.uk (J.M.); Nicola.Botting.1@city.ac.uk (N.B.); M.Cruice@city.ac.uk (M.C.); 2 Department of Communication Sciences and Disorders, Montclair State University, Montclair, NJ 07043, USA; boylem@mail.montclair.edu; 3 Speech Pathology, School of Medical and Health Sciences, Edith Cowan University, Joondalup, WA 6027, Australia; d.hersh@ecu.edu.au

**Keywords:** discourse, narrative, storytelling, connected speech, aphasia, theory

## Abstract

Discourse (a unit of language longer than a single sentence) is fundamental to everyday communication. People with aphasia (a language impairment occurring most frequently after stroke, or other brain damage) have communication difficulties which lead to less complete, less coherent, and less complex discourse. Although there are multiple reviews of discourse assessment and an emerging evidence base for discourse intervention, there is no unified theoretical framework to underpin this research. Instead, disparate theories are recruited to explain different aspects of discourse impairment, or symptoms are reported without a hypothesis about the cause. What is needed is a theoretical framework that would clarify the specific linguistic skills that create completeness, coherence, and complexity (i.e., richness) in discourse, and illuminate both the processes involved in discourse production and the reasons for breakdown. This paper reports a review and synthesis of the theoretical literature relevant to spoken discourse in aphasia discourse, and we propose a novel theoretical framework which unites these disparate sources. This framework is currently being tested as the foundation for Linguistic Underpinnings of Narrative in Aphasia (LUNA) treatment research. In this paper, we outline the novel framework and exemplify how it might be used to guide clinical practice and research. Future collaborative research is needed to develop this framework into a processing model for spoken discourse.

## 1. Introduction

Aphasia is a communication difficulty which can happen after a stroke or other damage to the brain, leaving a person with difficulties speaking and understanding others. Although each person experiences aphasia differently, a commonly expressed frustration is the reduced ability to communicate in everyday situations. Spoken discourse is key to everyday communication and so the detrimental effect that aphasia has on the form and content of discourse can have a huge impact on people’s everyday lives and those around them [1]. 

Discourse is often described in the theoretical literature as a unit of language larger than a single clause [2,3], forming a meaningful unit of language [4], and used for a specific purpose or function [5]. This characterization emphasizes the key role of spoken discourse in everyday interaction, for example talking about your day, describing a beautiful scene, or expressing your opinion about current affairs. In this paper, we focus on monologic discourse, although discourse occurs most often within a conversation, for example when one person takes a ‘turn’ for an extended period in conversation such as recounting an event when talking to friends. This focus on monologic discourse reflects the focus in recent aphasia assessment research [6,7] and in clinical practice [8]. 

Discourse has received a good deal of attention in aphasia research in recent years, most commonly in the form of monologues, as noted above. People with aphasia describe discourse as a *treatment* priority [9], although the evidence base largely addresses *assessment*. The assessment research literature describes people with aphasia as having trouble in a broad range of areas in the production of discourse. The evidence indicates that discourse can be impaired at a number of different linguistic levels: the language that speakers use; the information that speakers communicate; and the links that speakers are able to convey between pieces of information [6,7,10]. Therefore, there is a need to assess and treat different discourse and linguistic levels, and an emerging body of work describing multilevel assessment and treatment bears this out [11,12,13,14]. Whilst recent reviews revealed more than five hundred ways to measure discourse as reported in aphasia research over the last 40 years [6,7], thereby reflecting multiple approaches to discourse assessment, the key linguistic levels inherent in discourse production currently remain underspecified. The multiplicity of measures, coupled with the lack of a unifying underlying theoretical framework, leaves the onus on researchers and clinicians to select which aspects of discourse to measure and remediate without explicit guidance. 

There is a smaller body of evidence to support the treatment of spoken discourse. A recent review of spoken discourse treatment [15] highlighted the heterogeneity of treatment approaches aimed at improving discourse and to evaluate outcomes, even within this small field (25 papers, reporting on 127 participants). Moreover, eight of these 25 reviewed papers reported complex interventions, targeting multiple linguistic levels (discourse macrostructure, sentence and word). Best practice with complex interventions [16] is to develop them systematically, using the best available evidence and theory. However, only three of the reviewed papers explicitly reported their theoretical foundation, and they took diverse perspectives. A key theoretical basis for spoken discourse comes from linguistic theory, but there are multiple perspectives to draw on [17], which has created a challenge for researchers and has likely led to the paucity of theory in discourse treatment reporting. 

Theoretical frameworks and models are important in guiding clinical assessment to identify intact and impaired processes [18] which then allows a judgement to be made about which linguistic skill to target in treatment (both clinically and in research). In the broad field of psychology (encompassing psycholinguistics and aphasiology), a distinction is drawn between frameworks and models. Although both arise from theory, they differ in their purpose, such that a framework accounts for a phenomenon by providing a structure or outline consisting of descriptive categories (theoretical concepts or constructs) and the relations between them, while a model represents the system underlying the phenomenon [19,20,21]. For spoken discourse, however, there is little or no use of frameworks or models to underpin assessment and treatment planning or research.

This lack of theoretical underpinning in spoken discourse is not due to an absence of theory. There are numerous theoretical perspectives available to researchers and clinicians interested in discourse, which arise from diverse linguistic fields (including cognitive linguistics, psycholinguistics, sociolinguistics and pragmatics). These theories differ in the extent to which they are purely theoretical or empirically based, and so they consist of both frameworks and models, although there is very little explicit signaling of this difference in the reporting. There are isolated examples of spoken discourse assessment or treatment research referring to these linguistic theories, but overall, there is a lack of theoretical underpinning in this field of aphasiology and an absence of consensus about which theory or combination of theories to use. In a review of discourse assessment measures in aphasia by Pritchard and colleagues [7], it was found that although, post hoc, all of the measures surveyed could be broadly related to a clinical discourse processing model [22], none had been developed with explicit reference to it, or to any other theory. 

There is a need for a unified theoretical framework to move the field of spoken discourse research in aphasia forward. In the absence of such a framework, the evidence base is characterized by inconsistency, with research tending to report the surface characteristics of impaired discourse (the symptoms) rather than hypothesizing about how they might have arisen linguistically (the cause). Such hypothetical reasoning would require an underlying theoretical model. In the few instances where theory is referred to, disparate theories are recruited to explain different aspects of discourse impairment, thus limiting a synthesis of the evidence. The development and use of a theoretical framework would improve the relevance, comparability, and validity of the evidence base for discourse assessment and treatment in aphasia; furthermore, it would allow us to better synthesize, evaluate, adapt, and expand this field of research. Such a framework could shape clinical discourse practice and future research, and eventually result in a more fully specified processing model of spoken discourse.

In this paper, we describe the development of a novel theoretical framework for spoken discourse in aphasia. This was achieved through a review and synthesis of the existing theoretical literature, starting with the literature referenced in the evidence base for the assessment and treatment of discourse in aphasia and expanding to also encompass related literature from the field of linguistics. The goal of this review and synthesis was to create the novel, unified framework and to show how it can be used to describe spoken discourse difficulties in aphasia, and to inform novel treatment research. 

## 2. Materials and Methods

The methods outlined here constitute a type of metasynthesis, called metatheory, which is used to review and synthesize existing qualitative data (in this case, the discourse theory literature) for the purpose of theory building [23,24]. The methodology used for metatheory involves the analysis of theoretical perspectives, sources, and assumptions across multiple relevant studies [24]. An expansive literature search was conducted in order to identify the relevant studies. An expansive search contrasts with the exhaustive search that is necessary for systematic reviews of quantitative or qualitative data. An expansive search (recommended for metatheory review - such as this one) aims to find multiple presentations of relevant theoretical concepts, but the search is stopped when the information found is sufficient to clarify, explain, and inter-relate those concepts [25].

As a starting point, we searched the studies reviewed in four published systematic reviews of discourse assessment and treatment in the field of aphasiology [6,7,10,15]. The papers included in these reviews were examined to identify any underpinning theoretical frameworks or models related to spoken discourse. All explicitly mentioned theories (*n* = 7) were compiled into a list, which was then circulated to experts in the field (via social media channels). These experts (as defined by experience, reputation, or research track record in linguistics, aphasia, and/or discourse) were invited to comment on the completeness of the list of references derived from the reviews and asked to identify any missing frameworks or models. Nine experts responded, confirming the list and suggesting additional theories. The resulting list of theories (*n* = 10) is reported in the Results section.

The next stage in the metatheory procedure was to identify a representative publication relating to each of the identified frameworks for reading: this constituted the ‘source material’. Half of the list was read in full by the lead author, and the other half by a postdoctoral researcher. Each researcher read the complete publication and made notes about the following: concepts or constructs relating to discourse production; their description or definition; and any hypothesized inter-relationships between the concepts/constructs. This note making processes constituted the ‘data extraction’. Page numbers relating to where the data were extracted from were recorded. Following usual practice in psychology, a distinction was drawn between concepts and constructs. While both are considered to be descriptions of generalizable properties, patterns, and characteristics associated with a phenomenon (in this case with spoken discourse), a concept is a precise and measurable idea used to describe a pattern or property that can be observed (e.g., lexical diversity), whereas a construct is an abstract concept which is created to explain a pattern or property and which is hypothesized (e.g., lexical category). The extraction process revealed examples of both concepts and constructs in the source material, with no explicit acknowledgement of the difference. In order to check for the completeness and accuracy of the data extraction, each then read relevant extracts of the publications from the other half of the list, guided by the other researcher’s notes. Any differences in interpretation were discussed and resolved at this stage, and missing information was added to the notes. 

The extracted information contained in the notes (the ‘data’) was then synthesized and categorized using a qualitative content analysis approach [26,27]. This entailed coding the extracted concepts/constructs into a categorization matrix, using an iterative process which involved inductive content analysis of the notes as well as deductive reasoning. The aim was to separately categorize or to synthesize concepts/constructs based on the extracted description/definition and/or theoretical grounds (i.e., based on reasoning from first principles). All data extracted from the source material were coded for correspondence with the concepts identified in the matrix. A draft concept matrix was created by the first author and the postdoctoral researcher who completed the data extraction. This matrix was then refined by further consensus discussions, firstly with the wider research team (the other authors) and subsequently with experts in the field once again, this time through organized presentation and feedback sessions at three international conferences (2018-2019). The resulting matrix, judged to be the ‘best fit’ for the data, is outlined in the Results section (Table 1). 

**Figure 1:**
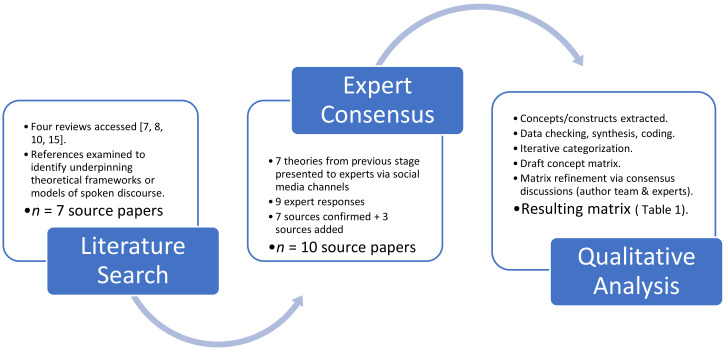
The metatheory process for the Linguistic Underpinnings of Narrative in Aphasia (LUNA) framework.

The methodological process described above is visually represented in Figure 1.

## 3. Results

**Figure 2:**
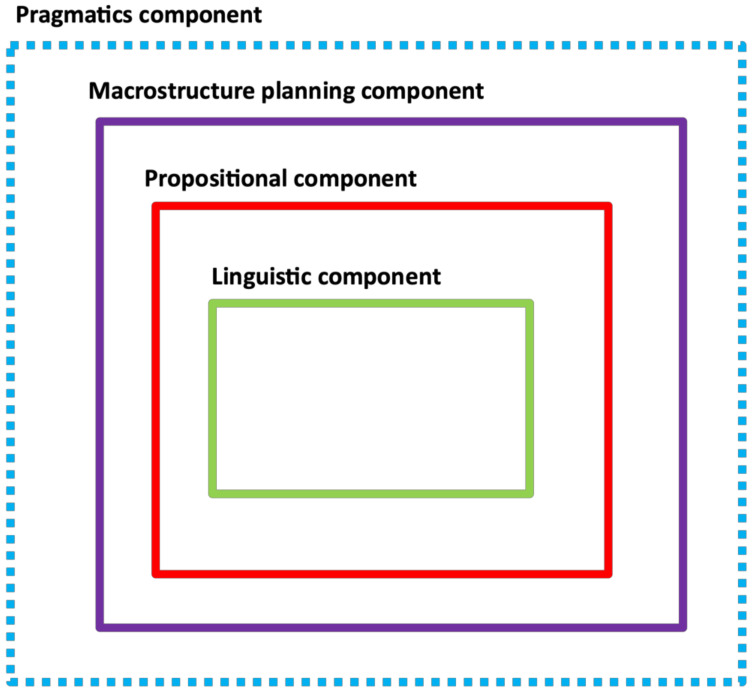
The LUNA framework for spoken discourse see Section 3.2. for information about each component.

Ten publications were identified to represent the theories (frameworks and models) which emerged from the expansive literature search process and expert consultation. This source material spanned the fields of linguistics, cognitive linguistics, psycholinguistics, sociolinguistics, and pragmatics; and consisted of both books and journal articles. These 10 publications are by the following authors: Frederiksen and colleagues [28], Halliday and Matthiessen [29], Kintsch and van Dijk [30], Labov [31], Levelt [32], Rumelhart [33], Sherratt [22], Slobin [34], Sperber and Wilson [35], and Stein and Glenn [36]. The core concepts/constructs extracted from these 10 publications are categorized in Table 1 (above) and described in the text that follows, which highlights similarities and differences in the source material as well as the overall complexity of spoken discourse theory. The categorization and descriptive summaries then lead to the proposed unified framework (depicted later in this section, in Figure 2) that is structured around the core categories: pragmatic, macrostructure planning, propositional and linguistic. The next section briefly reviews the theories reported in the 10 publications.

### 3.1. Summary of Frameworks and Models and in the Source Material

The 10 publications report six frameworks and four models (Frederiksen; Levelt; Sherratt; Sperber and Wilson). These 10 theories can be grouped into eight distinct groups, each of which is described in a separate paragraph below. 

The framework proposed by Frederiksen and colleagues [28] is derived from a range of discourse types (e.g., narratives and procedural) and describes three main levels of discourse processing (macrostructure planning, propositional, and linguistic), proposed to operate sequentially in discourse production. At the macrostructure planning level, discourse macrostructure is generated or retrieved from memory, as an overall template or frame. Additionally at this level, the schematic macrostructure is enriched with informational detail (such as factual and descriptive information about the events, participants, and context of the discourse); this information must then be further organized in order to select, foreground, prioritize, or otherwise arrange informational components as required by the linguistic system. At the propositional level, a set of propositions is generated from the organized information produced earlier at the macrostructure planning level. The sequence of propositions is decided at this level, making use of principles of inference and coherence; propositions are also grouped to ensure that there are an easy number to understand in each subsequent linguistic structure (e.g., in each clause or in each story episode). At the linguistic level, clauses are generated, syntax is assigned, content words within sentences are specified, and cohesion is created (such as by the use of reference chains). 

To the framework described by Frederiksen and colleagues [28], Sherratt [22] adds a more explicit role for pragmatics at each level as well as hypothesizing processing (thereby creating a model). Firstly, she highlights the pragmatic purpose of telling a discourse, by noting that there will be an initial ‘input trigger’ such as a direct request for a narrative or procedure, an auditory discourse to be recalled, or a visually presented narrative (e.g., picture or video) to be described. To the propositional level of the model, she adds a pragmatic component which is used as one of the principles for organizing propositions; and to the linguistic level, she notes that pragmatic constraints will also influence linguistic formulation choices. In addition to the greater emphasis on pragmatics in this model, Sherratt also explicitly adds retrieval of information from long-term memory, including semantic and episodic information, to the macrostructure planning level, making clearer the role of other cognitive systems and structures in discourse production (such as remembering, evaluating, reasoning, and establishing cause/effect.) 

In his framework, Halliday (see [29], for example) emphasizes both the structure and function of language, thus highlighting the linguistic ways in which meaning is achieved in discourse. Halliday also emphasizes the choice inherent in the language system at each linguistic level, asserting that when we analyze the language of a discourse, we are revealing the meaningful choices that have been made [29]. He uses the phrase ‘clause as a message’ to also emphasize the propositional content of language and creates a taxonomy of ways in which the language of a discourse might be used to achieve different meanings. The meanings he highlights variously underline different aspects of language use: the pragmatic role played by language (the way it affects or reflects the relationship among interactants), the propositional content the language represents (the information conveyed); and the effects of linguistic form used (differential effects from the choice, for example, between declarative and imperative sentence structures). One effect of linguistic form that is especially emphasized in the Halliday framework is cohesion (see also [2]), which defines the meaning of relationships between certain linked words in discourse (for example between pronouns and a related proper noun). Although cohesion consists of semantic links, its form is lexical and structural, and therefore this approach emphasizes the linguistic. Overall, Halliday’s framework provides a taxonomy of methods for conveying meaning in discourse. The emphasis is linguistic, focusing on the content and form of language, but also referring to propositional information and pragmatic effect. This theoretical framework is descriptive rather than intending to model processing. 

Kintsch and van Dijk’s framework [30,37] focusses on comprehension of expository discourse, although they explicitly state that the processes they discuss are likely to form the basis for production as well as comprehension. The arrangement and effect of these processes will be different as a reflection of the differential demands of language production versus comprehension [38]. The basis of this framework is the idea that, in order to understand a discourse, a mental representation of the story content must be created through a series of cognitive processes. This model presents a full description of discourse comprehension, but arguably its most distinctive contribution to this theoretical field is in its conception of the cognitive processes underlying propositional information. The authors posit mental representations which consist of propositional information, organized and synthesized into an essential macrostructure (or ‘gist’). In order to derive the gist from a text, three main types of processing are used: propositional, local coherence (meaning links between propositions), and global coherence. This processing results in a mental representation of the macro-structural properties of the discourse. 

Labov’s framework [31] describes the resulting structure of discourse, rather than the processing needed to produce it. In his search for authentic, natural language for his sociolinguistic studies of sound change, he analyzed personal narratives about a frightening situation. His analysis identified the following six recurring elements: abstract, orientation, complicating action, resolution, coda, and evaluation. Labov proposed that these elements are core to many different types of narratives, not just narratives of fear, and later work revealed similar narrative elements in, for example, oral memoirs, traditional folk tales, and narratives of everyday life. Similarly, Stein and Glen [36] identified seven elements of narrative discourse (setting, initiating event, internal response, internal plan, attempt + resolution, direct consequence, and reaction) and Rumelhart [33] identified ten elements (setting, episode, event, reaction, internal response, overt response, attempt, application, preaction, consequence). Although the elements are labelled slightly differently in these ‘story grammars’, there is a great deal of overlap in terms of the units of information that are being described. In Labov’s work, in addition to the findings about recurring macrostructural elements, there is also reference to some aspects of propositional content. For example, he distinguishes ‘free clauses’, which contain information that can occur anywhere within the story, from ‘narrative clauses’, which contain information that needs to occur within a particular place or a particular sequence in the discourse. He also describes some linguistic ‘devices’ which can be used to achieve evaluation, including adverbs, conjunctions, manner adverbs, and comparatives.

Levelt [32,39] provides a processing model to characterize the stages of speech production, incorporating pragmatic, macrostructure planning, propositional, and linguistic levels. The model begins with a ‘discourse model’ which incorporates pragmatic information (such as context and assumed shared knowledge) and cognitive information (such as information or knowledge from long-term memory). These two sources of information come together through macro-planning, which is “the process by which the speaker decides what to say next” [32] (p. 90), a process which involves planning an overall structure, which monitors reference and controls the discourse focus by guiding attention from topic to topic. This information is fed into a propositional level of processing that Levelt calls ‘the conceptualizer’, where micro-planning of the discourse occurs. At this stage, a propositional unit called a ‘pre-verbal message’ is created to turn pragmatic and cognitive components into the conceptual conditions that activate lexical items [39], thus creating the propositional format that will facilitate access to the linguistic system. This same process of synthesis is referred to by Slobin [34] as ‘thinking for speaking’, who expands on this aspect of processing to describe how information is packaged differently for different languages. Slobin’s description of conceptualization processing can be used to explain some of the difficulties experienced by people with aphasia when trying to express their thoughts [38]. As part of the conceptualization process, meaningful connections are also established between propositions using principles of local coherence. At the linguistic level of Levelt’s model, propositions (and the links between them) are grammatically encoded, and words are accessed. 

Sperber and Wilson proposed Relevance Theory [35], which is a processing model for explaining how we express and recognize meaning in language, allowing the exploration of the linguistic and structural choices we make to express meaning in discourse. This model highlights the influence of both cognitive and pragmatic processes, through its two key principles: human cognition is geared to maximize relevance (the Cognitive Principle); listeners expect utterances to be as relevant as possible (the Communicative Principle). In discourse terms, this means that, at a pragmatic level, discourse should be constructed with consideration for the way people will interpret it; and, at a macrostructure planning level, structure and story content should be organized in a manner that facilitates finding the relevant interpretation. Together these principles guide the construction of a discourse, whenever there is a choice (for example between one word and another, between sentence structures and ways to link them, and in the overall structure of information), towards the option that best balances the cognitive effort of the hearer with the reward of finding the relevance of each part of the discourse.

As the summaries above show, the source materials refer to a range of concepts and constructs involved in the description of spoken discourse. Through the metatheory synthesis process, four categories were identified (linguistic, propositional, macrostructure planning, pragmatic) into which these concepts/constructs could be grouped. Each theory (framework or model) in the source material referred to concepts or constructs in one or more of these categories-the categories are cross-referenced with the 10 theories in Table 2 (any additional texts relating to the same theories that were consulted are added here, alongside the main text for that theory). On the basis of this synthesis, we created a unified theoretical framework in which each category is depicted as a core component underpinning spoken discourse-see Figure 1. The validity of these categories can be initially evaluated by using the framework to describe case example, to explain assessment findings, and to motivate treatment (as we do in Section 3.3, below), but for a more thorough evaluation, further empirical research is needed. 

### 3.2. Overview of the LUNA Framework for Spoken Discourse

The individual frameworks and models cover many of the concepts and constructs of spoken discourse commonly referred to in the aphasia literature. However, none of these theoretical perspectives covers all of the relevant concepts/constructs or covers them in sufficient detail to be clinically useful or to underpin assessment and treatment research. Also lacking was a clear account of the relationship of one concept/construct to another. 

Our unified framework (the LUNA framework, Figure 2) aims to address these concerns. We propose that discourse production involves four categories (hypothesized processing components). The first is ‘Pragmatics’. This acts as a ‘filter’ or overall influence on spoken discourse. Here, the speaker makes decisions based on environmental, interpersonal, and interactional factors. For example, the context in which the discourse is delivered must be taken into account (at home vs. an institutional setting) and the effect of different interlocutors (a familiar conversation partner vs. a stranger) must be considered. These factors will, for example, influence language formality and the degree to which shared knowledge can be assumed. Interactional factors involve monitoring what is said against the developing linguistic context. For example, the speaker has to be aware whether s/he is introducing a novel topic or building on what has already been said. The interactional purpose of the discourse must be understood: is it to inform or entertain, reassure or surprise? Abilities required by the pragmatic component include working memory, to monitor the environmental and linguistic context, and the Theory of Mind, in order to reflect on the informational needs of the conversational partner. Social awareness will also be required, so that judgements can be made about whether particular usage (slang, swearing) is appropriate for the context.

The ‘macrostructure planning’ component involves the creation of an organizational frame or macrostructure for the discourse. The literature suggests that this frame is often composed from recurring structural elements, for example, to set the scene, outline the developing events, resolve the narrative, and evaluate/react to it [31,33,36]. This, in turn, suggests that the macrostructure planning component may draw on familiar templates, rather than having to build structures ‘de novo’ on each occasion. However, online structural decisions will be required. For example, different genres (telling a narrative vs. describing a procedure) require different frames and the speaker will need to ensure that all key information is covered. Some information may also need to be given more prominence in the structure than others; for example, in response to the pragmatic and social context. Although cognitive skills are relevant to all components of the LUNA framework, there are a number of cognitive skills and structures that are of particular importance in macrostructure planning. In addition to working memory, access to episodic memory will be needed, for example, to recall relevant events. Planning will call upon executive function, as will the need to monitor what has and has not been conveyed in the developing narrative (prospective memory and working memory). 

The ‘propositional’ component is a prelinguistic organizational component that feeds into linguistic processing. This parcels the structural organization or macro- ‘plans’ into the micro- ‘plans’ for individual utterances. For example, orientation elements of the structure will map onto utterances that set the scene (‘This happened last year in Italy’), while event elements will require utterances that convey actions (‘I was sailing the boat’). Local decisions are needed about entities to include (or omit) from propositions, and their role. For example, one entity may play the role of agent in a proposition while another may be the theme, or the object that is acted upon. The sequence of propositions is also determined, according to information dependency relations. For example, this may follow the logical steps of a procedure, cause and effect relationships, or a chronology. The propositional component builds the meaning relationships of individual utterances, and so involves semantic processing. As covered in our overview of the literature, it calls upon what Slobin [34] describes as ‘thinking for speaking’ and Levelt [32] describes as ‘conceptualization’, which involves cognitive skills such as perspective taking, selection, organization, and attention, as well as linguistic skills such as lexical and sentence semantics skills. 

The ‘linguistic’ component translates each proposition into fully realized utterances. The syntactic form is constructed, and lexical access takes place. Phonological assembly occurs and the utterances are articulated. Linguistic skills in syntactic construction, lexical retrieval, and phonological processing are key to this component. These skills are familiar to clinicians and researchers working in aphasia, and there are other theoretical frameworks and models that can be used to describe and/or explain the hypothesized processing in this component.

Moving on from a description of the content of each component, to consider the structure of the components, the LUNA framework proposes a specific relationship between components. In line with two of the theoretical frameworks [22,28] reviewed above, the components of spoken discourse are not considered to be sequential. In the LUNA framework, the relationship between components is conceptualized and depicted using a ‘Russian doll’ analogy, whereby linguistic processes sit inside propositional processes which sit inside macrostructure planning and pragmatic processes. According to this analogy, each component has the potential to influence every other one. Thus, pragmatic features (such as social context) will influence the overall organization of the discourse, the composition of propositions and even linguistic factors, such as vocabulary and syntax. To illustrate the latter, a formal context will impose vocabulary preferences (‘gentleman’ vs. ‘bloke’) and may encourage the use of particular syntactic options, such as the passive voice. We envisage that feedback can occur both ‘upwards’ and ‘downwards’ between all components in the LUNA framework, such that each component from pragmatic through linguistic will feed into the next one but components can also exert an influence in the other direction through revisions and reshaping as the discourse evolves. This would allow, for example, a word-finding or sentence formulation difficulty in the linguistic component to trigger a restructuring of information in the propositional component so as to drive an alternative formulation (a different word or sentence structure). This, in turn, may have wider ramifications for the macro-structure of the discourse.

The proposed LUNA framework enables us to reflect on the possible impact of aphasia on discourse production. For people with aphasia, the most obvious site of difficulty will be in the linguistic component. Here, for example, word-finding or sentence processing impairments will limit discourse utterances. It has also been argued that aphasia can lead to difficulties in the semantic/conceptual processes required by the propositional component [38,40]. For example, individuals may be unable to process event roles, or map these onto semantic verb-argument structures. The interconnected nature of the framework means that ‘local’ impairments may have wide reaching consequences for discourse production. For example, failure in propositional or linguistic processing may derail the macro-structure, or at least make it difficult to realize that structure. Similarly, even if the speaker is sensitive to pragmatic factors, they may be unable to reflect such subtleties in their output. There is the further possibility that the multi-faceted nature of discourse production (reflected in the LUNA framework) prevents the speaker from realizing their full linguistic potential. For example, it is known that some people with aphasia have linguistic competencies that are not fully realized in their discourse in all contexts [41]. For example, some individuals who can produce discourse in constrained conditions, such as picture description, cannot do so when faced with a different kind of discourse task, such as recounting their day. It may be that discourse, with its many components, imposes a processing cost that cannot be met by these speakers (especially those with more severe aphasia). Conversely, there is also evidence that for some individuals with aphasia, less constrained discourse tasks can reveal a more nuanced pattern of competence and difficulty. For example, Dipper and colleagues [42] compared the discourse produced by healthy speakers and those with mild-moderate aphasia in a picture description and more open discourse task (answering questions related to quality of life). They found that the more open task generated more mental verbs for both healthy speakers and those with aphasia, revealing competence in the linguistic component of the LUNA framework. There were also differences between the groups, in that the open discourse task resulted in less complex sentence structures for speakers with aphasia only, revealing an impact of genre in the propositional component of the framework.

Problems with discourse may additionally arise from impairments in attention, memory, and executive functions that often accompany aphasia. Murray [43] demonstrated that people with aphasia performed significantly more poorly than a control group in these cognitive domains, and that attention deficits were correlated to language and communication status. In terms of discourse production, any involvement of attention, memory, or executive function would impair the generation of macrostructure and make it difficult to track whether all essential information was being conveyed, resulting in omissions or loss of coherence. 

### 3.3. Use of the LUNA Framework for Spoken Discourse

The overall aim in creating this new framework was to address a gap in the theoretical literature and to provide a structure to guide the assessment and treatment of spoken discourse in research and practice. Our literature synthesis suggested that none of the existing frameworks described all of the theoretical categories of spoken discourse in sufficient detail, nor fully addressed the relationship of one category to another. In the following sections, we exemplify ways in which the LUNA framework proposed here might address these gaps. Firstly, we provide two clinical case examples which are described using the framework. Next, we explore the complexity of spoken discourse assessment in aphasia, from the perspective of the proposed framework, and finally, we briefly outline a new treatment for personal narrative discourse (LUNA treatment, an investigation of which is currently in progress) which has been informed by this framework. 

#### 3.3.1. Clinical Case Examples

To exemplify the use of the LUNA framework, consider the following examples of discourse produced by speakers with aphasia. These case examples have been extracted, with the speakers’ consent, from discourse produced either in a research project undertaken by the authors or as part of clinical work with one of the authors.

**Case 1**: This is a speaker with moderate non-fluent aphasia, responding to the prompt, “can you tell me about something that happened on a holiday you’ve been on recently?”.

*Good er plane and er er went near it’s er near there and er me and Linda and the bloke says go to the road and get that and oh alright ok oh stupid sorry it was quite good it was two weeks and that was good it was brilliant oh you know people it was quite good it was brilliant it was quite good oh I’m sorry.*

The linguistic component of this discourse is characterized by a limited range of nouns and use of semantically light verbs; sentences are mostly single clause (e.g., ‘[it]’s [near there]; [it] was [quite good]); and there is some unclear use of pronouns (e.g., ‘it’s near there’, ’go and get that’). In the propositional component, there is evidence that the overall topic has been organized into propositions for language, as evidenced by the overall sequence of words used and the combination of words, even where they are not complete utterances (e.g., ‘went near’, ‘the bloke says go and get that’, ‘it was two weeks’), but difficulty accessing words has limited the ability to convey this. In addition, there is a lack of coherence as to what is being communicated, how it relates to the question, and how each utterance relates to the previous utterance. In the macrostructure planning component, the discourse does not have a clear macrostructure, although some of the expected elements of the orientation section are conveyed—introduction of the characters/participants (‘Linda’, ‘the bloke’), a location (‘it’s near there’)—and there are attempts to describe key ‘events’ (‘go to the road and get that’). In the pragmatics component, the key factor is that this discourse was produced in response to a question about a holiday and so the relevance of the informational content should be considered in this context. In this context, there are some aspects of the discourse that are clearly relevant (e.g., the word ‘plane’, the information that ‘it was two weeks’ and the evaluation that ‘it was good’, ‘it was brilliant’). Overall, though, the relevance of what is being communicated and why is unclear. 

**Case 2**: This is a female speaker with moderate conduction aphasia responding to the prompt, “tell me about when you had your stroke”.

*Well I I was on the on the fla … I was … I think I was teluk right here on the on the teliff in the feliff first. And um my brother was coming to come over to bazzit. And she was just gone me call me. And all of a sudden that’s … I did … that’s that’s all I know. I I I was … I he he and m and she wife t t came from they had to with a cop and get it in min to go the line. And um they had to ma they he went over and da t went went to … from the t tea the the the light through the kazh and … When he and my brother and his life come come came they come in the they they found it by uh sent us by the f floor in the … And that’s all I can remember. I ended up in the hospiter. That was it.*

The linguistic component of this discourse is characterized by incomplete and inaccurate word retrieval and use of a limited range of semantically light verbs; sentences are revised and abandoned, some complex sentence structures are attempted, but not successfully, and pronouns are used incorrectly or ambiguously (e.g., “and she was just gone”). In terms of the propositional component, there appears to be some organization of the topic into propositions as evidenced by the sequence of verbs and the word combinations (e.g., ‘I was on the X’, ‘I was coming to come over to X’, ‘she was just gone me call me’), but the problems with retrieving words and formulating sentences prevent them from being realized. The word and sentence impairments also compromise cohesion and coherence: it is not clear how (or if) an utterance relates to the previous utterance or to the overall topic. In the macrostructure planning component, some elements of the expected macrostructure are attempted, but not always achieved. For example, the sample seems to start with a setting, but we are not quite sure what the location actually was or why her brother was coming over. There are attempts to convey key events, but since the verbs “came” and “went” predominate, often without their respective arguments, it is not clear exactly what transpired. In the pragmatic component, some elements of the discourse are clearly relevant to the topic of her stroke (e.g., “they had to with a cop and get it in”, “they found it by uh sent us by the f floor”, “that’s all I can remember”, “I ended up in the hospiter”), while others are more ambiguous (e.g., “I think I was teluk right here on the on the teliff”). 

In each of the example cases above, the word-finding difficulties are notable, but this is not the case for all speakers with aphasia. There are people with milder aphasia who nevertheless experience difficulties producing spoken discourse, difficulties that might manifest as coherence and cohesion issues, as well as milder linguistic issues such as not being able to access the vocabulary that they were previously able to and having to rely instead on more rudimentary/more accessible vocabulary. The LUNA framework is useful for tracking these ‘higher-level’ difficulties, since it allows for consideration of the skills needed in the components of propositional, macrostructure planning, and pragmatic processing, as well as linguistic skills, and furthermore prompts us to look at the impact of one component on another. 

#### 3.3.2. Assessment of Spoken Discourse 

In the aphasia evidence base, there are numerous ways to assess discourse skills; however, it is not always clear what construct or skill each measure is evaluating, how each measure relates to another, nor how measures should be selected in clinical practice and research. Under consideration here are not discourse protocols or specific tools, but rather a metric for measuring aspects of discourse (such as lexical diversity or correctness) which we are referring to with the term ‘measure’. To illustrate the point about a lack of clarity in the use of measures, consider the 500+ discourse-related measures identified in two recent reviews of the assessment literature [6,7] with no guidelines about which measure to use with which clients and why. Typically, the objective of researchers and clinicians is to choose one or more discourse-derived outcomes that are representative of an element of a person’s spoken discourse skills. For example, assessing the proportion of complete sentences produced in a discourse would provide an assessment of sentence production skill and provide information related to the theoretical construct of syntax. 

This distinction between theoretical constructs and related skills is not one routinely made in the aphasia literature, and this has led to some lack of clarity about what it is that discourse measures are measuring. Amongst the mass of discourse measures in the aphasia literature, the following range of constructs have been addressed: macrostructure; coherence; cohesion; informativeness; semantic-conceptual content; syntax; lexical-semantic content; lexical form; and verbal productivity. This has involved the assessment of the following skills, amongst others: production of specific schemas, or story grammar elements; production of linguistic items (words, sentences) relating to the topic or gist of a discourse; production of sentences that relate to each other; production of reference chains, and chains of lexical cohesion; production of linguistic items (words, sentences) denoting relevant semantic-conceptual content; production of linguistic items (words, sentences) with the correct form (syntactic, morphological or phonological); and production of fluent/ productive speech. These skills have been assessed by various methods including counts, proportions, evaluations of error or incompleteness, rating scales, and in the case of productivity, by counts per unit of time (e.g., words per minute). It would be helpful to be able to map these constructs and skills onto a framework of spoken discourse so as to begin to highlight the similarities and differences between them (and to illustrate gaps and overlaps in the discourse measures available). This mapping process is attempted in the following sections (and depicted in Figure 2). 

The connections between the skills and constructs are complex, as demonstrated in the clinical case examples presented earlier. It is difficult, if not impossible, to uniquely assess many of the theoretical constructs listed above because of their interrelatedness and the feedback between components of the LUNA framework. For example, an assessment of the proportion of complete sentences would provide information related to the constructs of syntax, semantic-conceptual content, lexical-semantic content, and lexical form. Some measures assess a theoretical construct only partially, such as correct information units (CIUs, [44]), a measure used to assess the skill of word production and provide information about word-level informativeness, but which does not provide information about the informativeness of the discourse more broadly. In addition, there is often more than one way to measure the same skill, such as assessing sentence production skill using the proportion of complete sentences, a mean predicate-argument structure (PAS, [45]) score, or the proportion of treated sentence structures produced. Finally, there is complexity arising from the lack of theoretical frameworks underpinning discourse assessment, which leads to a lack of specification about what a measure is measuring and how it might relate to another measure. This makes it difficult for researchers and clinicians to decide which measure(s) to use in an assessment, and how impairment in the skill being assessed might affect multiple levels of discourse production. Whilst the unified theoretical framework proposed in this paper cannot solve all of these problems, it can provide a structure in which to organize these interrelated strands (constructs, skills, measures) and which may help identify gaps and overlaps. 

**Figure 3:**
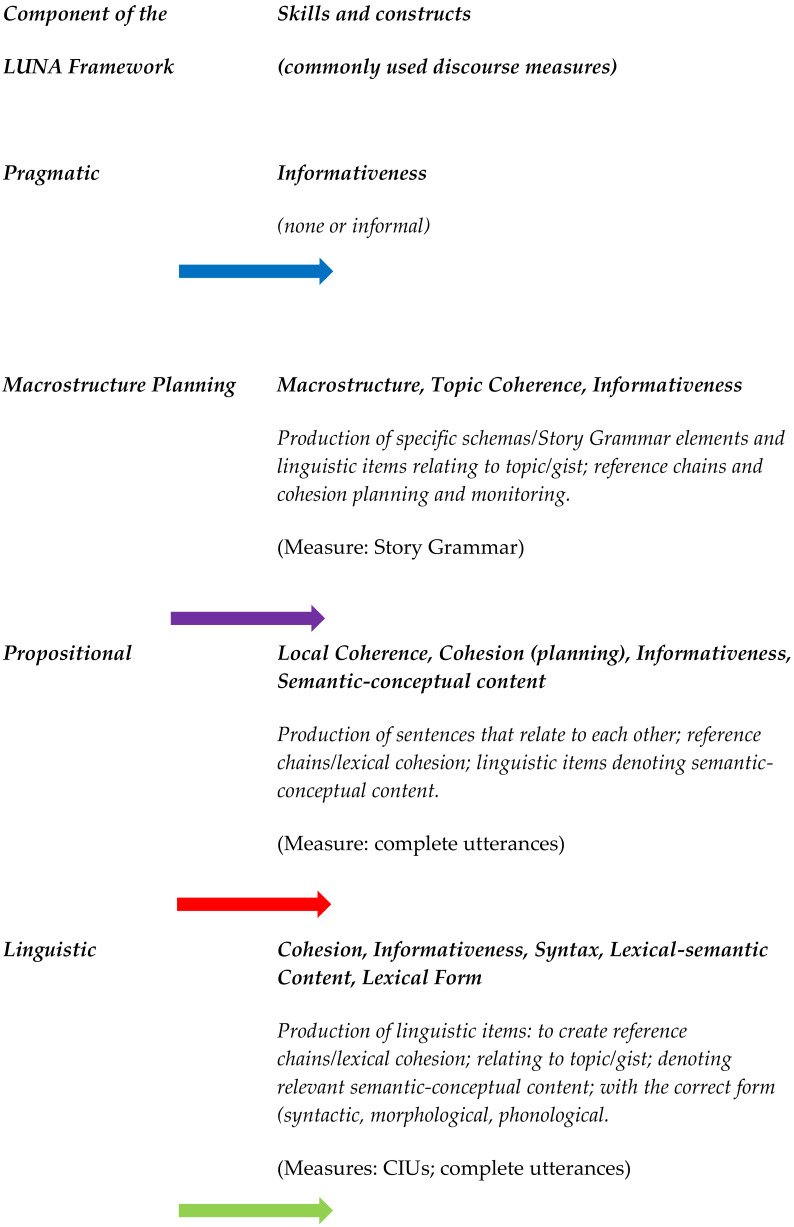
An example of the use of the LUNA framework: mapping skills, constructs and the three most commonly used discourse measures.

In Figure 3, below, we propose how the constructs and skills outlined above might be mapped onto the LUNA framework, along with three commonly used discourse measures (CIUs, complete utterances, and Story Grammar). Whilst it is beyond the scope of this paper to map all 500+ discourse measures onto this framework, this could be an informative exercise to pursue in a future endeavor. As Figure 3 shows, there is overlap in terms of which components of the LUNA framework best fit some of the theoretical constructs (e.g., informativeness and cohesion), discourse skills (e.g., production of reference chains) and measures (e.g., CIUs). This overlap might indicate that these constructs, skills and measures would benefit from further specification and decomposition. Alternatively, this overlap might be an intrinsic feature of the hypothesized interaction and feedback among the framework’s components. In the latter case, the aim of depicting these overlaps within the framework would be to map them precisely and to be aware of them in planning discourse assessment. There are also gaps in the aphasia literature identified through this mapping process, such as the lack of assessment of pragmatic skills relating to spoken discourse. Although speech and language therapists report informally assessing pragmatic skill using discourse [8], there are no commonly used formal tools to do this. Thus, mapping the measures onto the LUNA framework could provide direction for the development of needed assessment measures and discourage the proliferation of redundant measures.

Being able to situate a measure in the LUNA framework would also suggest targets for treatment, and might reveal links between performance in different components, as illustrated in the clinical cases above. For example, word-finding difficulties indicated by a low score in a linguistic measure such as CIUs [44] might occur alongside sentence level difficulties indicated by a measure that sits on the boundary between the linguistic and propositional components of discourse, such as mean PAS score [45]. In the macrostructure planning component, there may additionally be a lack of macrostructure indicated by a measure such as story grammar [14]; and difficulties in the pragmatic component might be indicated by a low score on a measure of listener judgement [46]. Rather than presenting these as the results from diverse assessments of different aspects of processing, the proposed framework unites them into components of spoken discourse. This, in turn, motivates and structures the search for a link between them and guides treatment design. 

#### 3.3.3. Treatment of Spoken Discourse

In proposing a unified theoretical framework, the aim was to guide both the assessment and treatment of spoken discourse in aphasia. In our recent systematic review of discourse treatment [15], we found 25 studies reporting on 127 participants with aphasia, indicating that discourse is an under-researched aspect of aphasia treatment. In the LUNA treatment research study currently in progress, we are aiming to develop a feasible and acceptable new discourse treatment for people with aphasia to improve their personal narratives, using the theoretical framework proposed here as a guide.

As noted earlier, LUNA stands for Linguistic Underpinnings of Narrative in Aphasia. It is a novel multi-level intervention which treats the linguistic skills required to tell effective personal narratives. In LUNA, the participant tells a personally chosen story about themselves, from which treatment targets are identified in the form of words, sentences, and elements of discourse macrostructure via linguistic analysis of the story transcript. Based on this analysis, potential lists of treatment targets are offered to the participant for discussion. The discussion aims to find out more about the purpose of the personally chosen story, the intended audiences for it, and ultimately to select which items (words, sentences, story elements) are to be treated. Treatment targets are then cumulatively addressed over 10 weeks.

The treatment in LUNA is thus organized into activities that target all components of the proposed theoretical framework. Word and sentence activities target the linguistic component; sentence activities simultaneously target the linguistic and propositional components; macrostructure activities target the macrostructure planning component; and the selection of treatment targets addresses the pragmatic component by considering the purpose and audience of the story. The cumulative approach ensures that words targeted at the start of the intervention also occur in the phrases, sentences and story structures targeted later, giving the intervention a strong coherence and allowing the person with aphasia to see the cumulative effect of each week’s work towards the improvement of the final story. The coverage of all components of the theoretical framework similarly strengthens the approach and ensures that all core elements underpinning successful spoken discourse are explicitly targeted in treatment.

## 4. Discussion

The aim of this paper was to review and synthesize the theoretical literature underpinning clinical approaches to discourse in aphasia. Theoretical frameworks and models are important both for research and practice. In clinical assessment, they can be used to identify intact and impaired components of processing and to inform treatment planning [18]. There is currently no unified framework routinely used to guide the treatment of spoken discourse, either clinically or in research. In a recent review of discourse treatment [15], most studies made no reference to any theoretical rationale for the methods they used. Where theory was explicitly mentioned, it related to just one level of language: discourse macrostructure [14,47,48]. The frameworks and models cited in these three treatment studies were part of the current review: Halliday and Hasan [2] for cohesion; and Labov [31], Rumelhart [33], and Stein and Glenn [36] for story grammar. However, discourse macrostructure is not the whole story. There are other levels of language involved in spoken discourse, such as words and sentences, and so there is a need to have an extended framework that covers all components of spoken discourse and which allows the identification of links between them. If our goal is to improve spoken discourse, then we must be aware that focusing treatment on a single discourse component (or single skill from within a component) might or might not affect other discourse components. Understanding how the skills and components are related can help us to predict which components of discourse production might change as a result of our treatment, to monitor for those changes and, if they do not occur, to implement treatment that focuses on the remaining impairments. The LUNA framework can provide a structured, systematic means for researchers seeking to understand how a treatment affects multiple components of discourse production and for clinicians seeking to ascertain whether their client’s overall discourse production has improved or needs further intervention.

Our review has resulted in the proposal of a novel unified framework outlining theoretically derived components of spoken discourse, with the intent of supporting assessment and treatment in research and practice. The framework is deliberately simple in its graphic presentation, which we hope increases its accessibility for a range of audiences including students, clinicians, and researchers. However, the concepts, constructs, and interrelationships that it seeks to encapsulate are complex. The next step in developing LUNA as a useful tool would be to conduct an ‘exhaustive’ review of the concepts, constructs, skills, and related measures in order to map some of the major connections delineated in the LUNA framework. In the previous sections, we aimed to emphasize the need for a unified theoretical framework that incorporates multiple components and to illustrate its uses. The need to consider multiple components (the four categories we propose here) in spoken discourse (and not just, for example, word production) is supported by a consideration of the evidence base, which indicates that aphasia affects discourse in a range of ways, including the language elements that speakers use, the information communicated, and the structuring of information in terms of linking ideas to each other and to the discourse macrostructure [6,7,10]. What is currently missing is an indication of how these difficulties overlap or otherwise relate to each other. The framework proposed in this paper should allow us to map discourse measures onto skills in a structured way, by organizing those skills into discourse components (linguistic, propositional, macrostructure planning and pragmatic), indicating areas where there are multiple measures that assess the same thing while other components lack any measure.

The evidence base for discourse treatment in aphasia would be strengthened by more treatment research underpinned by an explicit theoretical rationale, which would allow for an evaluation of what works and why [49]. Additionally, consensus about which theoretical framework to use would allow for a more systematic approach to assessment and treatment.

There are limitations in the current study that should be considered. Although the expansive literature search used here is the appropriate method for theory-building [25], it allows for the possibility that relevant literature has been omitted from the review. Whilst we can be confident that there were no omissions of theory from the field of aphasia assessment and treatment research, since the search method here (inspection of reference lists of papers included in systematic reviews) was exhaustive, we cannot be so sure about relevant theories from wider fields. However, as Finfgeld and Johnson [25] point out, the aim of an expansive search is to find enough source material to fully explicate concepts/constructs and the interrelationships between them. Another limitation is that the review and synthesis presented here was able only to propose a theoretical framework to guide future work, and not a processing model. As noted in the Introduction, a distinction is commonly drawn between frameworks and models. Both arise from theory although they differ in purpose: a framework describes a phenomenon whereas a model explains it. There is a consequent difference in the methods used for each approach, with frameworks arising largely from deductive (theoretical) methods and models additionally needing inductive (empirical) data to validate them [20]. A clear example of model use in assessment and treatment planning (and research) at the single-word level is the Psycholinguistic Assessments of Language Processing in Aphasia (PALPA) model [21]. Here, the data from test results can be used to generate clear hypotheses about strengths and impairments with words; and then treatment aims, methods and tasks can be selected or devised to address a client’s specific area of lexical difficulty. A next important step, requiring future collaborative research, would be to validate the framework with empirical findings in order to build a more detailed processing model of spoken discourse, including a consideration of the dynamic nature of the brain.

## 5. Conclusions

The review and synthesis presented here resulted in a novel unified theoretical framework for spoken discourse. Our aim in proposing the LUNA framework is to bring order and structure to the aphasia discourse assessment evidence base, and to provide a roadmap for emergent aphasia discourse treatment research. The objective is to improve discourse treatment for people with aphasia by clarifying the specific linguistic skills needed to create completeness, coherence, and richness in their discourse. Just as discourse is fundamental to everyday communication, we believe that theory is fundamental to moving the field of aphasia discourse research forward.

## Figures and Tables

**Table 1:** Categorization matrix for data extracted from the spoken discourse source material.

Category	Pragmatic	Macrostructure Planning	Propositional	Linguistic
List of the constructs and concepts extracted from the source material	Situational and external influences on discourse Context Interpersonal factors Interactional factors	Organization Structure Gist Story content Macro-structure, frames, genres Semantic memory ^1^ Episodic memory Macro-planning Coherence	Pre-linguistic Micro-planning Conceptualization Thinking for speaking Propositional information Utterance planning Sequencing Semantic content Sentence Semantics Information dependency relations Clausal planning Cohesion	Language Syntax Lexical semantics Lemma and lexeme Phonology

^1^ Whilst the distinction between semantic and episodic memory is controversial, we have retained it here because it reflects the content of the source material. Halliday, Kintsch, Frederiksen and Sherratt all use the term to distinguish two sub-systems of long-term memory: ‘semantic’ for factual knowledge and ‘episodic’ for recall of events.

**Table 2:** Source material mapped against four categories of concept/construct in spoken discourse-the shaded cells indicate which sources contain information relating to each category.

	Pragmatic	Macrostructure Planning	Propositional	Linguistic
Frederiksen and colleagues [28]				
Halliday and Matthiessen [29] Halliday and Hasan [2]				
Kintsch and van Dijk [30] van Dijk and Kintsch [37]				
Labov [31]				
Levelt [32] Levelt and Schriefers [39]				
Rumelhart [33]				
Sherratt [22]				
Slobin [34]				
Sperber and Wilson [35]				
Stein and Glenn [36]				
